# Optimal reconstruction methods after distal gastrectomy for gastric cancer: a protocol for a systematic review and network meta-analysis update

**DOI:** 10.1186/s13643-023-02445-5

**Published:** 2024-01-06

**Authors:** Zhou Zhao, Hancong Li, Xiangcheng Pan, Chaoyong Shen, Mingchun Mu, Xiaonan Yin, Jing Liao, Zhaolun Cai, Bo Zhang

**Affiliations:** 1 Department of General Surgery, Gastric Cancer Center, West China Hospital, Sichuan University, Chengdu, Sichuan 610041 China; 2 Department of Gastrointestinal Cancer Center, Chongqing University Cancer Hospital, Chongqing, China; 3 Department of General Surgery, Division of Thyroid and Parathyroid Surgery, West China Hospital, Sichuan University, Chengdu, Sichuan China; 4 Evidence-Based Pharmacy Center, West China Second University Hospital, Sichuan University, Chengdu, China; 5 Department of General Surgery, Division of Gastrointestinal Surgery, West China Hospital, Sichuan University, Chengdu, Sichuan China

**Keywords:** Billroth I, Billroth II, Gastric cancer, Network meta-analysis, Reconstruction, Roux-en-Y

## Abstract

**Background:**

Distal gastrectomy (DG) is a commonly used surgical procedure for gastric cancer (GC), with three reconstruction methods available: Billroth I, Billroth II, and Roux-en-Y. In 2018, our team published a systematic review to provide guidance for clinical practice on the optimal reconstruction method after DG for GC. However, since then, new evidence from several randomized controlled trials (RCTs) has emerged, prompting us to conduct an updated systematic review and network meta-analysis to provide the latest comparative estimates of the efficacy and safety of the three reconstruction methods after DG for GC.

**Method:**

This systematic review and network meta-analysis update followed the PRISMA-P guidelines and will include a search of PubMed, Embase, and the Cochrane Library for RCTs comparing the outcomes of Billroth I, Billroth II, or Roux-en-Y reconstruction after DG for patients with GC. Two independent reviewers will screen the titles and abstracts based on predefined eligibility criteria, and two reviewers will assess the full texts of relevant studies. The Bayesian network meta-analysis will evaluate various outcomes, including quality of life after surgery, anastomotic leakage within 30 days after surgery, operation time, intraoperative blood loss, major postoperative complications within 30 days after surgery, incidence and severity of bile reflux, and loss of body weight from baseline.

**Ethics and dissemination:**

The review does not require ethical approval. The findings of the review will be disseminated through publication in an academic journal, presentations at conferences, and various media outlets.

**INPLASY registration number:**

INPLASY2021100060.

**Supplementary Information:**

The online version contains supplementary material available at 10.1186/s13643-023-02445-5.

## Introduction

### Description of the condition

Gastric cancer (GC) is a significant cause of cancer-related mortality, accounting for 5.6% of all cancer cases and 7.7% of cancer-related deaths worldwide, with a mortality number of 768,793 in 2020 [1]. Although multidisciplinary gastric cancer care has improved survival rates through chemotherapy, radiotherapy, and molecular targeted therapy [2], radical surgical resection remains the only definitive therapeutic treatment for individuals with localized GC [3, 4]. For most GC in the lower two-thirds of the stomach, distal gastrectomy (DG) is the recommended surgery. However, the choice of reconstruction method after DG is still controversial, with various methods introduced since Billroth conducted the first subtotal gastrectomy in 1881 [5]. Billroth I (B-I), Billroth II (B-II), and Roux-en-Y (R-Y) are all valid reconstruction methods [6].

### Description of the intervention

B-I and B-II reconstructions are widely used in Asia due to their procedural simplicity [7], but they also have complications. For instance, Billroth I reconstruction may increase anastomotic tension and the incidence of fistula, despite having physiological advantages [8]. Billroth II reconstruction avoids anastomotic tension to some extent, but it cannot be applied to all patients because it may lead to postoperative alkaline reflux gastritis, dumping syndrome, esophagitis, and anastomotic ulcers [9–11]. R-Y reconstruction prevents alkaline reflux gastritis and reflux esophagitis, and reduces anastomotic tension [12]. Despite these advantages, patients who have undergone R-Y reconstruction often experience Roux stasis syndrome [13–15]. Therefore, finding an appropriate digestive tract reconstruction method to reduce postoperative complications and improve quality of life is crucial.

### Why it is important to do this review

Our team conducted a Bayesian network meta-analysis to investigate the comparative evidence of B-I, B-II, and R-Y reconstruction for patients with GC after DG, combining direct and indirect comparisons [16]. In 2019, two additional meta-analyses also investigated this question [17, 18]. However, they included observational studies as well as randomized controlled trials (RCTs), potentially leading to bias. Furthermore, Min et al. conducted a meta-analysis on this topic in 2022 but only searched the PubMed database [19]. Relevant publications may have been missed as a result. Moreover, these studies [17–19] did not evaluate the confidence in the findings from network meta-analyses. As several RCTs have been published in recent years, potentially providing clinical efficacy evidence for the three reconstruction methods after DG for GC, we plan to conduct a systematic review and network meta-analysis update to expand our previous work [16] and provide more robust clinical evidence. In this updated review, we will adjust primary outcomes of interest to include quality of life after surgery and major postoperative complications within 30 days after surgery. Additionally, we will update the literature search as well as include a broader range of study types and adjust the effect sizes.

## Method

This review will update a previously published systematic review and meta-analysis by our team.

### Protocol and registration

This protocol is written in accordance with the Preferred Reporting Items for Systematic Review and Meta-Analysis Protocols (PRISMA-P) (Supplementary file 1) [20]. Any amendments to this protocol will be recorded in an updated version of the INPLASY registration. The updated work will be conducted in accordance with the PRISMA guidelines and the standard methodology recommended by the Cochrane Collaboration [21, 22].

### Inclusion criteria

#### Types of participants

We will include studies with patients who had histologically proven gastric cancer located in the distal stomach and without evidence of distant metastasis.

#### Types of studies

We will include randomized controlled trials (RCTs) and quasi-experimental studies, wherein the method for allocating participants to different interventions was not strictly random (e.g., by date of birth, day of the week, month of the year, medical record number, or order of inclusion in the study). Sensitivity analyses will be conducted, excluding quasi-randomized trials, to assess the robustness of the results. The studies considered for inclusion will have been published before February 1st, 2024.

#### Types of interventions

We will include RCTs that compare two or more of the following reconstruction methods after DG for GC: B-I, B-II, or R-Y reconstruction.

#### Types of outcome measures

The primary outcomes will be as follows:

1. Quality of life after surgery (measured by validated questionnaires) (at least six months).
2. Major postoperative complications within 30 days after surgery (grade III to V according to Clavien‐Dindo Classification) [23].

The secondary outcomes will be as follows:

1. Operation time
2. Hospital stays
3. Incidence of anastomotic leakage within 30 days after surgery.
4. Incidence and severity of bile reflux according to endoscopic examination or according to the criteria of the original studies (at least 6 months).
5. Loss of body weight (kg) from baseline (at least 6 months).

### Exclusion criteria

1. Studies with overlapping data.
2. Full-text articles are not available after exhaustive searches to locate the texts.

### Information sources and search strategy

One reviewer will perform a systematic literature search in PubMed, Ovid Embase, and the Cochrane Central Register of Controlled Trials databases for eligible studies from their inception. Medical subject headings terms (Mesh) combined with text words and synonyms will be performed in our search course. In addition, the manual search and reference search will be performed to enlarge the search range. The reference list of relevant publications will also be hand-searched to identify additional potential studies. A sample PubMed search strategy is described in detail in online Supplementary file 2. In PubMed search strategy, we combined the subject-specific strategy with the sensitivity and precision-maximizing version of the Cochrane highly sensitive search strategy for identifying randomized trials, 2008 revision [21].

### Study selection and data extraction

Two reviewers will independently screen titles and abstracts to exclude obvious irrelevant reports, using the reference management software Endnote X20. They will then independently examine the full text of all potentially eligible articles. Disagreements will be resolved through a team discussion. We will record the excluding reasons and generate a PRISMA flow diagram for the network meta-analysis [24].

Two reviewers will extract data independently using a standardized data collection form. The following data will be extracted from the included studies: the first author, publication year, country, study design, participant characteristics, inclusion/exclusion criteria, intervention details, and outcome data. If multiple studies are conducted on the same subjects, only the study with the highest methodological quality, the most complete results, or the most recent published date will be included [25]. The authors of the trials will be contacted to provide missing data if needed. Disagreements will be resolved through a team discussion.

### Dealing with missing data

For missing data, we will attempt to obtain more information from the original authors. In the absence of a reply, we will try to calculate the data through the available coefficients according to the *Cochrane Handbook for Systematic Reviews* [21]. For continuous outcomes, SDs will be estimated by stand errors, p values, or CIs, depending on how the original research is provided. Otherwise, SDs will be evaluated based on the median or IQR. The potential impact of these missing data will be assessed by sensitivity analysis. We will not exclude the studies with insufficient data, we will describe these studies in the supplementary and explain the reasons why they will not be included in meta-analysis.

### Risk of bias assessment

Two reviewers will independently assess the quality and risk of bias of the included studies by using the Cochrane Risk of Bias Tool (RoB I) in the following domains [26]: random sequence generation, allocation concealment, blinding of participants and personnel, missing outcome data, selective outcome reporting, and other bias. The risk of bias in each domain, as well as the overall risk of bias, will be rated as ‘low risk’, ‘high risk’, or ‘unclear’. Disagreements will be resolved through a team discussion. When inadequate information is available from the studies to rate a risk of bias item, we will contact the corresponding authors for additional information.

### Data analysis

#### (1) Geometry of the network

We will use Hiplot (available at hiplot-academic.com) to draw network plots to describe and present the geometry of types of interventions including B-I, B-II, and R-Y reconstruction for patients with GC after DG for each outcome. Each node will be a treatment. An edge will connect two nodes when at least one trial compares the two corresponding treatments. The size of the nodes will be proportional to the number of patients, while the weight of each edge will be proportional to the number of studies per treatment comparison.

#### (2) Assessment of transitivity

We will use a narrative summary to describe the characteristics of each included study. To assess transitivity, we will compare the distributions of baseline participant characteristics across studies and treatments to confirm that they are parallel among different comparisons. If differences are found, then these will be addressed in subgroup and sensitivity analyses.

#### (3) Network meta-analysis

We will apply all NMAs using a Bayesian Markov chain Monte Carlo framework and fitted in R [27] using the *gemtc* [28], and *rjags* [29] package. For NMAs, we will report the estimated weighted mean differences (WMDs) for continuous outcomes, whereas the estimated risk ratios (RRs) for dichotomous outcomes, as well as the 95% credible interval (95% CrI). This is in preference to the odds ratio (OR) since ORs (when interpreted as RR) can markedly exaggerate the effect size when event rates are high [30–32]. To rank the efficacy for each intervention, we will calculate the ranking probabilities for all treatments, the surface under the cumulative ranking curve (SUCRA) [33].

#### (4) Heterogeneity assessment

When multiple trials are available for comparison, heterogeneity within each pair-wise comparison will be assessed using the Cochran Q test with *I*^*2*^ statistic [34]. We will interpret the *I*^*2*^ statistic according to the guidelines in the Cochrane Handbook [30, 35] as follows: 0 to 40% might not be important; 30 to 60% may represent moderate heterogeneity; 50 to 90% may represent substantial heterogeneity; 75 to 100% represents considerable heterogeneity. The Chi^2^ test will be interpreted where a *P* value less than or equal to 0.10 will indicate evidence of statistical heterogeneity. For network meta‐analysis, we will assess statistical heterogeneity in the entire network by considering the magnitude of the between‐study variance parameter (Tau2) derived from the network meta‐analysis models. We will compare the magnitude of the heterogeneity variance with empirical distribution for dichotomous outcomes, as proposed by Turner 2012 [36]. Both pairwise meta-analysis and network meta-analysis (NMA) will be conducted using random-effects models [34].

#### (5) Assessment of inconsistency

We will use the node-splitting method, which involves splitting mixed evidence into direct and indirect evidence in each node for comparison, to assess inconsistency [37]. If a discrepancy is not found, this network meta-analysis can be considered to fit the consistency model. Otherwise, when a significant difference between direct and indirect evidence occurs, an inconsistency model will be utilized, and potential reasons for inconsistency will be discussed [38].

### Meta-biases and confidence in the findings from network meta-analysis

To assess small-study effects, we will visually explore the comparison-adjusted funnel plot for each outcome when at least 10 studies are available. The confidence in the findings from network meta-analysis will be evaluated based on the latest version of the Confidence In Network Meta-Analysis (CINeMA) web tool [39, 40] (available at cinema.ispm.unibe.ch). CINeMA is based on a methodological framework that considers six domains: within-study bias, reporting bias, indirectness, imprecision, heterogeneity, and incoherence [39].

## Discussion

This updated systematic review and network meta-analysis aims to comprehensively evaluate the latest evidence regarding the optimal reconstruction method for patients with GC after DG. Our analysis will consider various clinical outcomes such as quality of life after surgery and major postoperative complications within 30 days. Additionally, we will provide recommendations for future research directions in this area and highlight the clinical implications of our findings for informing treatment decisions.

### Supplementary Information

**Additional file 1: Supplementary 1.** PRISMA-P 2015 checklistR1.

**Additional file 2: Supplementary 2.** PubMed search strategy.

## Data Availability

Not applicable.
