# Supplementary material for: Optimal reconstruction methods after distal gastrectomy for gastric cancer: a protocol for a systematic review and network meta-analysis update
Source: Syst Rev. 2024 Jan 6;13:19. doi: 10.1186/s13643-023-02445-5 (PMC10770945; doi:10.1186/s13643-023-02445-5)
Supplement: Supplementary file 2 — Additional file 2: Supplementary 2. PubMed search strategy. [file 13643_2023_2445_MOESM2_ESM.pdf]

PubMed search strategy

#1 (((("stomach neoplasms"[MeSH Terms] OR ("stomach"[All Fields] AND "neoplasms"[All Fields]) OR "stomach neoplasm"[All Fields] OR ("gastric"[All Fields] AND "cancer"[All Fields]) OR "gastric cancer*"[All Fields]) OR ("stomach cancer*"[All Fields])) OR ("Gastric Neoplasm*"[All Fields])) OR (Gastric adenocarcinoma)) OR (Gastric carcinoma)

#2 "gastroenterostomy"[MeSH Terms] OR "gastroenterostomy"[All Fields] OR ("billroth"[All Fields] AND "ii"[All Fields]) OR "Roux-en-Y or Roux-Y"[All Fields]

#3 ((((((((randomized controlled trial [pt]) OR (controlled clinical trial [pt])) OR (randomized [tiab])) OR (placebo [tiab])) OR (drug therapy [sh])) OR (randomly [tiab])) OR (trial [tiab])) OR (groups [tiab])) NOT (animals [mh] NOT humans [mh])

#4 #1 AND #2 AND #3
